# Student performance and grading changes in a systems-based curriculum

**DOI:** 10.3402/meo.v19.23165

**Published:** 2014-01-09

**Authors:** Philip E. Cooles, Miscilda Harrigan-Vital, Agnes Laville

**Affiliations:** Department of Clinical Medicine, Ross University School of Medicine, Roseau, Dominica

**Keywords:** curriculum change, assessment, student study strategy

## Abstract

**Introduction:**

Changing from a conventional discipline-based, basic science medical school curriculum to an integrated systems-based curriculum, which was graded on overall performance not by discipline, was associated with a highly significant improvement in an externally administered comprehensive exam at the end of the 2-year basic science program. The reason for the improvement was unclear, but we hypothesized that it was partly due to a change in student study strategy. Anecdotal evidence suggested that students had changed their study habits to spend less time on previously harder-to-pass courses and more time on courses with previously lower failure rates. If this were so, performance in previously harder disciplines might have deteriorated, while performance in previously easier disciplines could have improved.

**Methods:**

We examined relative performance in the four discipline-based courses of Systemic Pathology, Microbiology, Pharmacology, and Introductory Clinical Medicine (ICM) in the fourth (and last) semester of the curriculum. We compared failure rates in these four courses for the three cohorts before the change with the three cohorts after.

**Results:**

Pharmacology and Microbiology which had failure rates over twice as high as Systemic Pathology and ICM in the conventional program before the curriculum change showed a deterioration in performance after the change with an approximately threefold rise in internal failure rate. In contrast, Systemic Pathology showed a dramatic improvement with a more than threefold drop in the failure rate, while the ICM clinical skills course, which was largely assessed by a practical exam in all 6 cohorts, showed no change.

**Conclusion:**

The improvement in student performance in an external assessment may be due, at least partly, to a change in the school's internal grading policy which led to a more appropriate balancing of student study strategies. Future studies on the effect of curriculum change should include an assessment of the impact on student study strategies.

## Introduction

While medical school curricula have traditionally been organized around disciplinary lines, integrated and systems-based approaches are gaining increasing favor (1). While these new models are thought to better motivate and stimulate students by providing additional meaning and context to the information presented, little empirical evidence has demonstrated improved outcomes. Ripkey and associates found no significant differences in students’ United States Medical Licensing Exam (USMLE) Step 1 scores based on the general type of basic science curriculum (2).

Located in Caribbean West Indies, Ross University School of Medicine (RUSM) has an allopathic medical program that each year admits three freshman classes of approximately 300 students. In 2010, RUSM reorganized its 2-year basic training from a discipline- to a systems-based curriculum. This revision entailed two major changes: first, by moving to a modular structure, efforts were made to better integrate content, optimize its timing, and avoid unintended duplication; second, students’ grades were based on their overall performance in modules rather than in individual disciplines. Thus, poor performance in one discipline could now be offset by strong performance in another. Lectures, learning objectives, professors, and exam questions remained largely unchanged.

Prior to the curriculum change, the last (fourth) semester of the 2-year basic science sequence consisted of four courses: Microbiology, Pharmacology, Systemic Pathology, and Introductory Clinical Medicine (ICM). Consistently, both Microbiology and Pharmacology had twice the failure rates of Systemic Pathology and ICM. Consequently, students were thought to spend comparatively more study time on the former two. Again, under the old curriculum, grades were assessed on students’ demonstrated knowledge of specific disciplines.

Along with internal modular-based examinations, RUSM continued to administer the National Board of Medical Examiners’ (NBME) comprehensive basic science subject exam at the end of the second year to gauge students’ preparedness for the USMLE Step 1 licensing exam. Thus, any change in student performance – due either directly or indirectly to curricular revisions, grading schemes, or other factors – should be reflected in this assessment.

Although it was not possible to identify unique sources of potential variance in exam scores, we hypothesized that students would show significant performance declines in Microbiology and Pharmacology and performance increases in Systemic Pathology and ICM.

We anticipated no significant changes in students’ NBME comprehensive exam scores.

The following reflects a cursory analysis and discussion of these questions.

## Methods

We examined students’ performance by discipline for the three (each) fourth-semester classes before and after the curriculum change and compared the observed ‘pre-revision’ failure rates in the four disciplines with ‘post-revision’ rates using the same grading policies.

All exam questions were in a single-best-answer format. In the modular exams, questions were distributed equally among disciplines. There were no changes in the total numbers of questions per discipline or the question format.

Prior to the grading change, 20% of students’ ICM performance was based on a practical OSCE-style exam. This component remained following the curricular revisions, but was now assessed separately from the systems modules (a separate requirement to pass this component was initiated). All data were de-identified per institutional review board approval of the study.

## Results

Compared with many schools, RUSM experiences variable enrollment numbers. So, while a total of 1,030 and 770 students, respectively, comprised each of the three-semester cohorts before and after the curricular revision, the general ‘profile’ of matriculants did not change. Indeed, mean Medical College Admission Test (MCAT) scores (23.2 vs. 24.3) and entering undergraduate grade point average (GPA) (3.17 vs. 3.23) did not vary significantly for the two groups.

As hypothesized, we documented a significant decline in students’ performance in Microbiology and Pharmacology. In fact, applying the pre-change cutoff point for passing, the rate of student failures in Pharmacology would have increased from 6.5 to 17% and from 6.4 to 14% in Microbiology. Concurrently, students’ performance in Systemic Pathology increased – with failure rates declining from 3.2 to 0.2%. However, performance in ICM remained virtually unchanged. This trend was consistent in all three post-change semesters.

While we had no firm hypothesis regarding learners’ performance on the NBME comprehensive shelf exam, the first class of students in the new systems-based curriculum scored significantly (*p*=≤0.001) higher than the previous three cohorts: 68.6 versus 62.8%, respectively. Subsequent cohorts showed this same pattern, suggesting that the change was probably real, and not due to a simple observation effect (3).

## Discussion

While the impact of transitioning our basic science curriculum (and grading scheme) from a discipline-based to a modular systems-based model remains unclear, marked changes in students’ performance – including a sustained improvement in students’ NBME comprehensive shelf examination scores – were noted. It is certainly possible that integrating the teaching led to improved student learning and, hence, improved exam performance. Yet, accompanying the curricular revisions was a significant change in grading policy: Rather than assessing performance by discipline (as was previously done), we now assessed performance comprehensively via the system modules.

So, if improved teaching and/or learning were responsible for the increased NBME exam scores, we might have expected a similar rise in our internal exams. However, this was not the case: Disciplines with the greatest failure rates before the curricular change rose dramatically, while the subjects with the lowest failure rates improved further. The drop in student performance in Microbiology and Pharmacology internal exams, coupled with a rise in Systemic Pathology, suggests that this change in grading policy may have played some role.

Anecdotal accounts indicate that students’ study strategies may have changed in response to the new curriculum. Before the curricular change, students appeared to have focused effort on courses they felt most likely to fail – conversely, paying less attention to those they viewed as comparatively easy. With the subsequent grading changes, however, students could now maximize their chances of passing the semester by preparing for all disciplines equally. Consequently, this may have led to further performance improvements in Systemic Pathology – at the expense of historically more difficult disciplines. Unfortunately, since we have no direct measure of study time, we cannot empirically verify this possibility.

In some ways, the ICM course may have served as a sort of ‘curricular control’, since the lack of change in students’ performance may be explained by the fact that 20% of this grade was based on an OSCE-style practical exam. After the curriculum change, this exam was administered ‘outside’ the modular system on a pass–fail basis, while the written exam questions continued to contribute toward the modular grade. Thus, since the passing requirement remained unchanged, students’ study time for the practical exam may have been less affected.

The possibility that grading policy changes either directly or indirectly resulted in increased student performance, then, is plausible, since assessment is often thought to drive learning (4) and can influence student study patterns (5). The potential relevance of grading policy may also add credence to other studies which have failed to show marked performance effects of planned curricular changes (2, 6, 7).

In any medical school curriculum, student study time is a finite resource that learners must allocate accordingly. As a result, there is a sort of built-in ‘competition’ between courses and/or departments for students’ attention and motivation. One way of vying for this, it appears, is to make exams more difficult – something arguably more easily done in discipline-based curricula. Indeed, as Muller and colleagues point out, a loss of academic control is sometimes a concern with integrated curricula (8).

In summary, the reasons for the observed changes remain conjectural, and our analysis of this curricular change perhaps raises more questions than it answers. Nonetheless, the take-home message is that assessment is a powerful driver of student effort and learning, and should be appropriately balanced in the interests of the various stakeholders. Future studies on the effects of curriculum change on student performance should consider the specific roles of grading policies and study strategies on how students learn and allocate their time.
